# Underestimation of the number of COVID-19 cases, an epidemiological threat

**DOI:** 10.1017/S0950268822001728

**Published:** 2022-11-08

**Authors:** Alexandre Vallée

**Affiliations:** Department of Epidemiology-Data-Biostatistics, Delegation of Clinical Research and Innovation (DRCI), Foch Hospital, Suresnes 92150, France

The study of Judson *et al*. reported that the diversity and differences among COVID-19 reporting systems in African countries revealed international lessons for data reporting during pandemics [1]. Most African countries reported event detection data, with total cases, deaths and recoveries. Most countries also have sophisticated, routine situation reports or online dashboards which may be updated in real-time providing additional risk assessment and response data. As of 24 February 2021, the African region accounted for 2.5% of cases and 2.9% of deaths reported globally [2]. Nevertheless, some countries showed differences in reporting healthcare capacity and preparedness data [2].

However, based on the study of the World Health Organization (WHO) published in April 2022, more than 60% of Africans have been infected with COVID-19 since the beginning of the COVID-19 worldwide pandemic. That is 97 times more than the officially observed confirmed cases [3]. According to this study, between January 2020 and December 2021, 800 million Africans had been infected by SARS-CoV-2, while only 8.2 million cases had been reported. The WHO considers that the underestimation of actual infections occurred everywhere, but that it was greater in Africa.

Globally, true worldwide infection rates were 16 times higher than the confirmed cases [3]. The risk of emergence of more deadly variants, stronger than the immunity acquired during previous infections cannot be ruled out, so it is imperative to call for an intensification of testing. The WHO study showed that exposure to the virus rose from 3% in June 2020 to 65% in September 2021, with sharp increases after the arrival of both Beta and Delta variants.

Challenges and developments will strongly depend on the progress of both national and worldwide vaccination strategies, the initiation and spread of variants of concern and public health programmes focused on non-pharmaceutical responses [4–6]. Different studies have shown that 45% of the world's inhabitants have been infected by September 2021, but the WHO statutes comparisons were complex because the studies covered different time periods.

The seventh wave of COVID-19 observed in Europe may also be greatly underestimated with the number of non-reports or the preference in the general public for self-tests without confirmation by RT-PCR. In France, more than 26 million people have contracted the virus since the beginning of the pandemic, 23 million cases in Germany, more than 15 million in Italy and nearly 12 million in Spain. These data seem to be greatly underestimated. In Africa, only 16% of the African population is vaccinated (so only 209 million people), in Europe over 75% [7]. But in these two geographical areas, the vaccination rate remains too low to counteract the COVID-19 pandemic.

The WHO shows that vaccinating a population which has already been infected gives higher protection than that offered by infection-induced immunity alone. It seems imperative to continue public health policies to promote the vaccination of populations to obtain herd immunity. A dual policy of intensive testing to assess the epidemic curves as accurately as possible will make it possible to better prevent the next steps of the COVID-19 pandemic and to better appreciate epidemiological scenarios with strong predictive performance [8].
